# Astrocyte Activation in Neurovascular Damage and Repair Following Ischaemic Stroke

**DOI:** 10.3390/ijms22084280

**Published:** 2021-04-20

**Authors:** Adjanie Patabendige, Ayesha Singh, Stuart Jenkins, Jon Sen, Ruoli Chen

**Affiliations:** 1 Brain Barriers Group, School of Biomedical Sciences and Pharmacy, University of Newcastle, Callaghan, NSW 2321, Australia; Adjanie.Patabendige@newcastle.edu.au; 2 Priority Research Centre for Stroke and Brain Injury, and Priority Research Centre for Brain & Mental Health, University of Newcastle, Callaghan, NSW 2321, Australia; 3 Hunter Medical Research Institute, New Lambton Heights, NSW 2305, Australia; 4 Institute of Infection & Global Health, University of Liverpool, Liverpool L7 3EA, UK; 5 School of Pharmacy and Bioengineering, Keele University, Staffordshire ST5 5BG, UK; a.singh@keele.ac.uk; 6 School of Medicine, Keele University, Staffordshire ST5 5BG, UK; s.i.jenkins@keele.ac.uk (S.J.); j.sen@keele.ac.uk (J.S.); 7 Neural Tissue Engineering: Keele (NTEK), Keele University, Staffordshire ST5 5BG, UK; 8 Clinical Informatics and Neurosurgery Fellow, The Cleveland Clinic, 33 Grosvenor Square, London SW1X 7HY, UK

**Keywords:** stroke, astrocyte, neurovascular, neuroprotection, neurotoxicity, neurorestoration, neuroinflammation, secretion

## Abstract

Transient or permanent loss of tissue perfusion due to ischaemic stroke can lead to damage to the neurovasculature, and disrupt brain homeostasis, causing long-term motor and cognitive deficits. Despite promising pre-clinical studies, clinically approved neuroprotective therapies are lacking. Most studies have focused on neurons while ignoring the important roles of other cells of the neurovascular unit, such as astrocytes and pericytes. Astrocytes are important for the development and maintenance of the blood–brain barrier, brain homeostasis, structural support, control of cerebral blood flow and secretion of neuroprotective factors. Emerging data suggest that astrocyte activation exerts both beneficial and detrimental effects following ischaemic stroke. Activated astrocytes provide neuroprotection and contribute to neurorestoration, but also secrete inflammatory modulators, leading to aggravation of the ischaemic lesion. Astrocytes are more resistant than other cell types to stroke pathology, and exert a regulative effect in response to ischaemia. These roles of astrocytes following ischaemic stroke remain incompletely understood, though they represent an appealing target for neurovascular protection following stroke. In this review, we summarise the astrocytic contributions to neurovascular damage and repair following ischaemic stroke, and explore mechanisms of neuroprotection that promote revascularisation and neurorestoration, which may be targeted for developing novel therapies for ischaemic stroke.

## 1. Introduction

Globally, stroke is a leading cause of death and disability, causing over 5.5 million deaths in 2016 [1]. Ischaemic stroke, caused by a blocked artery leading to the brain, is the most common form of stroke, accounting for approximately 87% of all strokes [1]. In cerebral ischaemia, two distinct zones can be identified in the affected brain region: the ischaemic core and the penumbra. In the ischaemic core, brain cells die due to a lack of glucose and oxygen because of the loss of blood flow. In the penumbra where the blood flow is reduced, peri-lesional neural tissue is biochemically and metabolically compromised and is at risk of further damage and/or necrosis, but it also represents a potentially salvageable region of tissue, if appropriate treatment measures are available [2,3]. Reperfusion remains the only immediate treatment option following ischaemic stroke. *Thrombolysis* by recombinant tissue plasminogen activator [4] and clot removal by mechanical thrombectomy [5,6] are effective in acute stroke management [7,8]. However, these reperfusion strategies are only applicable to a small percentage of patients due to a short therapeutic time window, contra-indications and the costs associated with establishing the infrastructure to deliver these treatments [9]. There lies an opportunity for further neuroprotective intervention [10]. Neuroprotection is not an alternative to the treatment techniques of thrombectomy and thrombolysis; rather, it seeks to restrict injury to the brain parenchyma following an ischaemic insult by preventing salvageable penumbral neurons from dying. The concept of neuroprotection has shown promise in experimental studies, but has failed to be translated into clinical success [10,11]. A recent notable example is that of the eicosapeptide nerinetide that interferes with post-synaptic density protein-95, an excitatory neuronal protein; this showed promise in pre-clinical studies, but failed to show any benefit in human stroke trials [11]. One of the reasons for the failures of acute neuroprotective strategies is that they have largely targeted neurons. However, the brain comprises various other cell types, such as glial cells (astrocytes, oligodendrocytes and microglia), endothelial cells and pericytes, all of which influence neuronal function and survival [12]. These cells have not been sufficiently studied as therapeutic targets in ischaemic stroke, but understanding of their post-ischaemic behaviours, both harmful and beneficial, is increasing.

Glia are more numerous than neurons, except within the cerebellum, where human brain neurons outnumber glia by 4.3:1 [13,14]. Throughout the rest of the central nervous system (CNS), the ratio of glia to neurons in humans [14,15] ranges from 1.7:1 in the cerebral cortex [12], to 11.4:1 in the midbrain and hindbrain [10]; it is 17:1 in the thalamus [16]. Glial cells have important roles in brain homeostasis as well as in the development and maintenance of the blood–brain barrier (BBB) [17]. They can have either protective or detrimental effects on neurons following ischaemic stroke [18,19,20,21,22,23,24].

**Figure 1:**
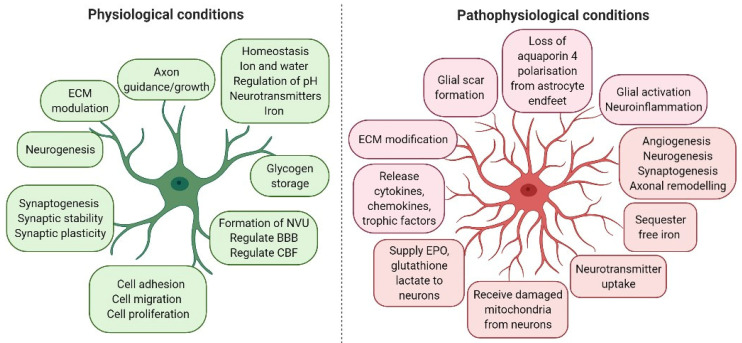
The diverse functions of astrocytes during physiological and pathophysiological conditions. Astrocytes have several important roles in the healthy and diseased brain. They are the main housekeeping cells of the brain, and can exert either protective or detrimental effects on neurons during pathophysiological conditions. Astrocytes are essential for the development and maintenance of the BBB, homeostasis of the brain microenvironment, cerebral blood flow regulation, neurotransmitter uptake, synaptogenesis, neurogenesis and release of neurotrophic factors and energy supply to neurons. Astrocytes also play a major role during pathophysiological conditions. Neuronal survival heavily depends on astrocytes. For example, neurons do not survive if neighbouring astrocytes are lost during an ischaemic stroke. Cerebral ischaemia leads astrocytes to change their morphology and function, causing astrocytes to become reactive, thereby producing several proinflammatory modulators and participating in glial scar formation. Their neuroprotective roles include clearing glutamate from synaptic regions, secretion of neurotrophic factors and promotion of angiogenesis, neurogenesis, synaptogenesis and axonal remodelling. Figure created with BioRender.com.

Astrocytes are the most common glial cell type, and probably the most common cell type in the CNS. A large number of astrocytes survive after a stroke and exert a regulatory effect in response to ischaemic stroke. Thus, astrocytes have become a potential treatment target for stroke [18,19,20,21]. Astrocytes fulfil many important roles in both healthy and injured brains (Figure 1). These include maintaining ion and pH homeostasis in the CNS; promoting the synthesis and removal of neurotransmitters; providing glucose supply and antioxidant defence; regulating synaptic activity by producing various cytokines, chemokines, growth factors and metabolites; supporting neurons; regulating cerebral blood flow (CBF); and supporting BBB formation and function [25].

Astrocytic morphological and functional characteristics are altered under pathological conditions, a process termed “reactive astrogliosis” (Figure 1). Astrogliosis is characterised by cellular hypertrophy, proliferation and increased expression of glial fibrillary acid protein (GFAP). It has been demonstrated that the increase in GFAP-positive cells in astrogliosis results not from the generation of new astrocytes, but rather from an increase in GFAP synthesis and a condensation of glial filaments in pre-existing cells, leading to more readily detectable GFAP by immunostaining [26]. In addition, S100β, a marker of astrocyte activation, is released from astrocytes during brain injury. More recent studies and genetic profiling of astrocytes led to the identification of another marker of astrocyte activation-aldehyde dehydrogenase 1 family member L1 (Aldh1L1) [27]. ALDH1A1 is a marker of astrocytic differentiation during brain development and correlates with better survival in glioblastoma patients [28]. Activated astrocytes also upregulate other structural and adhesion molecules, extracellular matrix (ECM) components and inflammatory chemokines and cytokines. This can result in a positive feed-forward loop, because the secretion of inflammatory cytokines further activates local cells.

Given the diverse functions of astrocytes, the purpose of this review is to summarise the role of astrocyte activation in the degeneration and regeneration of the neurovasculature following ischaemic stroke, and explore the roles of astrocytes in neuroprotection, neurotoxicity, neuroinflammation and neurorestoration following cerebral ischaemia.

## 2. Astrocyte Activation in Neuroprotection, Neurotoxicity and Neurorestoration

Astrocytes are essential for cell–cell communication in the neural tissue, being directly in contact with neurons, oligodendrocytes, microglia, endothelial cells and pericytes. In the human brain, astrocytes may have up to two million synapses within their domains. Their cellular processes enwrap synapse terminals and modulate neuronal activity [29]. Astrocytes form a “tripartite synapse” in the brain, and are a physical barrier to prevent neurotransmitters from diffusing away from the synapse [19]. They protect neurons during ischaemia by clearing glutamate from synaptic regions via glutamate transporters. Glutamate is converted by glutamine synthetase into glutamine that is shuttled back into the presynaptic terminal and re-used for glutamate synthesis [19]. Over 80% of glutamate transporters, especially excitatory amino acid transporter 2 (EAAT2, also known as glutamate transporter 1, GLT-1 in rodents), are located on astrocytes, making astrocytes the main site of glutamate uptake in the brain [30]. Glutamate uptake by astrocytes in ischaemia is essential for neuroprotection, as it terminates glutamate’s effects as a neurotransmitter, but also prevents extracellular glutamate levels from reaching excitotoxic levels [31].

Pellerin and Magistretti [32] reported that glutamate additionally stimulated glycolysis (i.e., glucose utilisation and lactate production) in astrocytes, and postulated the astrocyte-neuron lactate shuttle (ANLS) hypothesis which defines the strong metabolic association between astrocytes and neurons. The ANLS hypothesis states that astrocytes serve as a “lactate source”, whereas neurons serve as a “lactate sink” [32]. Lactate shuttling consists of astrocytic production of lactate through glycolysis or glycogenolysis; the lactate is then exported via the astrocyte-specific monocarboxylate transporters (MCT1 and MCT4) and taken up by neurons via MCT2 [33]. However, Bak et al. [34] claimed that neurons were well equipped to metabolise glucose in an activity-dependent manner, and preferred glucose over lactate. The emerging role of astrocytes has helped in settling this debate in favour for ANLS hypothesis [35,36].

In the brain, glycogen is stored mainly in astrocytes, the utilisation of which can sustain periods of high neuronal activity during hypoglycaemia [37]. Astrocyte glycogen plays an important role in maintaining neuronal survival during conditions of hypoglycaemia in vitro [38] and in vivo [39]. A part of the glucose that enters the astrocyte is converted into glycogen before entering the glycolytic pathway, despite this being energetically unfavourable compared to classical glycolysis [40]. In addition, astrocytes receive damaged mitochondria from neurons for mitophagy, and deliver healthy mitochondria to neurons [41]. Enhancing mitochondrial energy production is key to re-establishing the function of brain cells after stroke.

Astrocytes have been shown to promote neuronal survival by enhanced synthesis and release of antioxidants such as glutathione (GSH), and increased expression of transcription factors such as NF-E2-related factor 2 (Nrf2) [42]. Astrocytes are the main source of GSH in the CNS, and the concentration of GSH in astrocytes is twice that of neurons [43]. Neurons co-cultured with activated astrocytes had a 1.7-fold fold increase in GSH concentration compared with neurons cultured alone [44], and the viability of neurons was significantly enhanced by Nrf2-dependent enhancement of glial GSH synthesis and release [45], strongly supporting the profound antioxidant activity of astrocytes, which protects brain cells from death during strokes.

Astrocytes form highly interconnected networks via gap junctions, which are clusters of channels made from connexins (Cx30 and Cx43) located in astrocyte endfeet. Gap junctions mediate intercellular communication and solute movement between astrocytes [46], and regulate extracellular potassium concentration [19]. Cx43 expression has been shown to increase following hypoxia/ischaemia injury [47], and abnormal opening of these channels can lead to excitotoxicity and increased inflammation due to uncontrolled release of glutamate and ATP, and a Ca^2+^ overload [48,49]. Inhibiting gap junctions either protects neurons from death by restricting the flow of neurotoxic metabolites or increases the susceptibility of co-cultured neurons to glutamate cytotoxicity [50,51,52]. Li et al. [47] have demonstrated the potential to target Cx43 as a treatment strategy for improving stroke outcomes. Inhibition of Cx43 in a neonatal rat model reduced active astrogliosis and cerebral infarct volume, and improved functional recovery [47].

Liddelow et al. [53] termed the two subtypes of reactive astrocytes “A1” and “A2”. They found that A1 reactive astrocytes were induced by IL-1α, tumour necrosis factor (TNFα) and C1q secreted by activated microglia. These astrocytes have few physiological functions but contribute to the death of neurons and oligodendrocytes. One of the most upregulated genes in A1 reactive astrocytes is complement component C3. In contrast, this is not upregulated in A2 reactive astrocytes, which upregulate many neurotrophic factors and thrombospondins, and promote neuronal survival and tissue repair [54]. Therefore, C3 can be used as a marker to differentiate reactive astrocyte types. Reactive astrogliosis can generate neurotoxic mediators such as S100β. S100β at low doses can be neuroprotective, but during ischaemia, high levels released by astrocytes can be neurotoxic. A clinical trial targeted reducing secretion of S100β from astrocytes in ischaemic stroke patients produced a favourable trend in reduction of the National Institutes of Health Stroke Scale (NIHSS) that should be confirmed in a future clinical trial [55].

In subacute and chronic stages, cerebral ischaemia recovery relies on profound neurorestoration processes, including angiogenesis, neurogenesis and synaptogenesis [19]. Astrocytes promote the secretion of various neurotrophic factors, such as nerve growth factor (NGF), brain-derived growth factor (BDNF), neurotrophin 3 (NT-3), erythropoietin (EPO), vascular endothelial growth factor (VEGF), epidermal growth factor (EGF), insulin growth factor (IGF), glial derived neurotrophic factor (GDNF) and basic fibroblast growth factor (bFGF). These trophic factors promote angiogenesis, neurogenesis, axonal remodelling and the growth/survival of neurons and oligodendrocytes during ischaemia [56,57]. Astrocytes may also function as support cells in the subventricular zone where neural progenitor cells reside [58].

Astrocytes express growth factors and signalling molecules (such as members of the Jagged/Notch and WNT signalling pathways) that regulate stem cell proliferation and differentiation, and modulate neuroinflammation. Notch signalling influences astrocyte morphology, and in ischaemic lesions, regulates the proliferation of reactive astrocytes [59]. In human astrocytes, hypoxia has been shown to upregulate the WNT signalling pathway [60]. For human astrocytes cultured in hypoxic conditions, hypothermic treatment inhibited WNT signalling, possibly indicating a reason why hypothermia has not reliably demonstrated efficacy as a stroke therapy [60]. The composition of the ECM in the CNS, much of which is astrocyte derived, also plays a critical role at certain sites in controlling stem cell fate, maturation and survival [61].

## 3. Astrocytes Modulate Cerebral Blood Flow, Angiogenesis and the Blood–Brain Barrier

Astrocytes make extensive contact with blood vessels and play an important role in regulating CBF, in addition to primary CBF regulators such as small arteries and arterioles, which either dilate or contract under the influences of multiple complex physiological control systems (i.e., cerebral autoregulation). Glutamate, a major neurotoxic neurotransmitter in ischaemic stroke, activates metabotropic glutamate receptors (mGluR) in astrocytes, leading to an increase in intracellular Ca^2+^ and the synthesis of arachidonic acid (AA). AA, along with the metabolites derived from AA generation in astrocytes such as prostaglandin E2 (PGE2) and epoxyeicosatrienoic acids (EETs), dilate blood vessels [62]. In addition, the release of K^+^ from astrocytes may also contribute to vasodilation [63], whereas secretion of 20-hydroxyeicosatetreanoic acid (20-HETE) from astrocytes constricts vessels [64,65].

Following cerebral ischaemia, astrocyte swelling is one of the earliest responses, which is mainly due to (i) translocation of aquaporin 4 (AQP4) to the cell surface, facilitating water influx down an osmotic gradient (ionic oedema) [66], and (ii) increased uptake of glutamate and lactate [67]. AQP4 knockout mice subjected to ischaemia showed a 35% reduction in brain oedema [66]. Hypoxia-induced translocation of AQP4 is influenced by Ca^2+^, calmodulin (CaM) and protein kinase A (PKA), and oedema-related pathology can be reduced through pharmacological inhibition of Ca^2+^, CaM or PKA [66]. In the ischaemic brain regions, swollen astrocytes compress cerebral vessels, leading to further reduction of CBF [68].

Hypoxia inducible factor (HIF), an important transcriptional factor that regulates hypoxia-responsive genes, is implicated in the ischaemic brain [69]. HIF activation in the penumbra promotes angiogenesis, which enhances the transport of oxygen and glucose to the brain in subacute and chronic phases. However, it does not contribute to the recovery in the acute phase, as capillary restructuring requires at least a week [70]. VEGF, a HIF downstream gene, is upregulated in the penumbra upon onset of cerebral ischaemia [71]. In mammals, the VEGF gene consists of five subtypes, VEGF-A/B/C/D/F, including placental growth factor. VEGF-B mediates embryonic angiogenesis in myocardial tissue, and VEGF-C mediates lymphangiogenesis. The primary molecule that is associated with endothelial cell proliferation in cerebral ischemia is VEGF-A. VEGFs bind to VEGF receptors, namely, VEGFR-1, 2 and 3 [71]. The increase in VEGF-A and VEGF-receptors begins as early as 2–4 h after the onset of stroke and lasts for at least 28 days [72].

Angiogenesis is the growth of new blood vessels from existing vessels, typically in response to hypoxia. Angiogenesis encompasses coordinated remodelling of the basal lamina matrix with endothelial cells to generate new blood vessels. The extent of angiogenesis within the penumbra of ischaemic stroke correlates with the patients’ survival time [73]. VEGF’s role in neuroprotection is less understood, but its ability to influence neuron survival is noted [71]. Increasing VEGF has been shown to increase neural proliferation markers such as 5-bromo-2′-deoxyuridine (BrdU) in the hippocampus [73]. Zhang et al. [74] showed that VEGF mediated an increase in CBF that maintained the penumbral blood supply. Nevertheless, VEGF can activate matrix metalloproteinases (MMPs) such as MMP9 and downregulate tight junction proteins such as claudin-5 and occludin, leading to BBB disruption during the acute phase of ischaemic stroke [75], leading to haemorrhagic transformation and further brain cell death [76].

The critical importance of astrocytes in the induction and maintenance of BBB structure and function has long been established [77,78,79,80,81]. Confocal microscopy studies have shown that brain endothelial cells are surrounded by the perivascular endfeet of astrocytes that contain rosette-like whorls of filaments. Multiple endfeet from the same astrocyte can interact with several endothelial cells, and an endothelial cell may be surrounded by endfeet from several astrocytes [82]. Several studies have provided a great deal of information to support the role of astrocytes in upregulating many BBB features, including low paracellular permeability/functional tight junctions [83,84,85,86], transporters [87,88,89] and enzymes [90]. In addition to astrocytes, pericytes seem to play an important role in orchestrating the proper formation of the BBB and the NVU. Pericytes extend their processes along and around pre-capillary arterioles, capillaries and post-capillary venules, and may have different morphological and functional features depending on their positions along the vascular tree [91].

**Figure 2:**
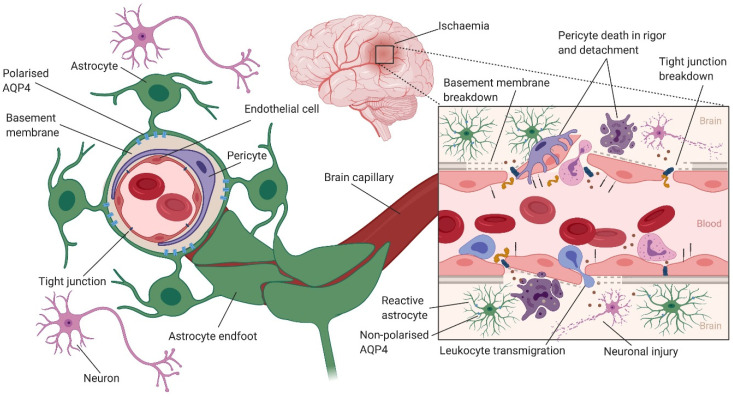
Blood–brain barrier (BBB) dysfunction following ischaemic stroke. (1) Simplified structure of the neurovascular unit (NVU) comprising brain endothelial cells, astrocytes, pericytes and neurons. Brain endothelial cells form tight junctions between them that control the movement of molecules through the paracellular pathway by showing size and charge selectivity, thereby forming a selective barrier between the brain microenvironment and the systemic circulation. Pericytes partially cover the endothelial cells and are embedded in the basement membrane. They are spread discontinuously along the microvessel, and maintain the barrier properties of brain endothelial cells, regulate capillary diameter, cerebral blood flow and angiogenesis. Perivascular endfeet from multiple astrocytes ensheath endothelial cells, allowing intricate cell–cell communications to help maintain the BBB phenotype of brain endothelial cells. Astrocytes secrete soluble factors that are important for the development and maintenance of the BBB. In addition, astrocytes contribute to brain water homeostasis via aquaporin 4 (AQP4) water channels, which are expressed on the endfeet of astrocytes. (2) The schematic on the right demonstrates BBB dysfunction due to cerebral ischaemia. Loss of tissue perfusion due to ischaemic stroke leads to neuronal injury and death. This causes an increase in the release of proinflammatory mediators that activates endothelial cells, astrocytes and pericytes. The resulting neurovascular inflammation disrupts tight junctions, leading to paracellular leakage and cerebral oedema. During ischaemia, AQP4 lose their polarisation from astrocyte endfeet. A dual role is played by AQP4 in cytotoxic and vasogenic oedema, where AQP4 deficiency leads to a reduction in cytotoxic oedema and improved neurological outcomes, and vasogenic oedema is increased when AQP4 is knocked down. The activated brain endothelium attracts leukocytes, which results in increased transcytosis across the BBB and neuroinflammation. This proinflammatory environment leads to changes in astrocyte morphology: “reactive astrogliosis”. Reactive astrocytes can secrete proinflammatory mediators such as IL-1α, IL-6 and TNF-α that cause further BBB disruption and neuronal injury. In addition, ischaemic stroke causes pericyte detachment and death in rigor, which can lead to vessel constriction and the no-reflow phenomenon. Figure created with BioRender.com.

Increased BBB permeability following ischaemic stroke has been shown to have a complex biphasic profile [92,93]. An early opening of the BBB within hours of ischemia is followed by a refractory phase, and then a second opening in 72 h [92,94]. Using transgenic mouse models and two-photon time-lapse microscopy, Knowland et al. [95] visualised structural tight junction protein localisation and BBB permeability following transient middle cerebral artery occlusion (MCAO). They demonstrated that an early (within 4–6 h after stroke) increase in BBB permeability was associated with upregulation of endothelial transcytosis, while the delayed (2–3 days after stroke) increase in BBB permeability was due to the remodelling and disassembly of tight junction proteins [95] (Figure 2).

BBB breakdown leads to cerebral oedema [96,97] and haemorrhagic transformation [98,99], which are common complications of ischemic stroke that can impact the outcomes of these patients with potentially serious and life-threatening consequences [100]. BBB breakdown is the main cause of vasogenic oedema, where water and plasma proteins enter the brain interstitial space, leading to brain tissue swelling as the brain capillaries behave like fenestrated capillaries. However, cytotoxic oedema does not lead to brain tissue swelling. Rather, cells of the CNS, particularly astrocytes swell following CNS injury (e.g., ischaemic stroke). During ischaemic stroke, a fall in cellular ATP levels leads to the inhibition of ATP-dependent transporters (e.g., Na^+^/K^+^ ATPase). The resulting influx of osmolytes such as Na^+^, which generates an osmotic force, drives an influx of water into the cells, causing cellular swelling [101,102]. Astrocytes in particular play a major role in cytotoxic oedema via the water channel AQP4, as discussed in detail in the next section.

## 4. The Role of Astrocytic Aquaporin 4 in Cerebral Oedema Formation

Aquaporins (AQPs) are small integral membrane proteins (MW ∼ 30,000) that mainly facilitate water transport across cell membranes in response to osmotic gradients, and are found in several tissues, including the brain, kidneys and lungs [103]. Fourteen AQPs have been identified in humans and rodents, with at least eight of these having been shown to be directly involved in water transport [104]. AQP1, 4 and 9 are expressed in the brain, but only AQP1 (located in the apical membrane of choroid plexus epithelial cells) and AQP4 (highly expressed on astrocyte endfeet; in the subpial astrocyte processes and the basolateral membranes of ependymal cells; and in subependymal astrocyte processes), are critically important in regulating brain water flux [104,105]. In addition to water transport, a subgroup of AQPs known as aquaglyceroporins (AQP3, 7 and 9) transport glycerol, along with small polar solutes, ions and various gases [106,107,108]. Furthermore, AQP4 has been shown to be involved in astrocyte migration, glial scar formation, neuroinflammation and extracellular K^+^ uptake [109].

Oedema is a major and often life-threatening consequence of stroke [101]. Cytotoxic oedema has been shown to be primarily mediated by AQP4, as the water is transported through AQP4 located in astrocyte endfeet into the CNS with the BBB intact [110,111]. However, vasogenic oedema is independent of AQP4, and water enters the CNS through intercellular spaces due to BBB breakdown [111]. Frydenlund et al. [112] have shown that the expression level of perivascular AQP4 pool is subject to regional-specific temporary changes after transient MCAO in mice. This has important implications for cytotoxic oedema formation and dissolution, as perivascular AQP4 allows bidirectional water flow, and therefore is most likely to be the rate-limiting step for both water influx and efflux after ischaemic stroke [112]. In support of this, AQP4-deficient mice had reduced cytotoxic brain oedema and improved neurological outcomes 24 h after permanent MCAO [113]. Further work has shown that AQP4 knockout mice had decreased infarct volume, neuronal cell death and neuroinflammation, and had improved long-term outcomes after transient MCAO compared with wild type mice [114]. However, AQP4 knockout mice had increased water accumulation at three and seven days after transient MCAO compared with wild type mice [114]. This suggests that vasogenic oedema due to reperfusion rather than cytotoxic oedema is responsible for this increase in water content and impaired clearance. Therefore, AQP4 inhibitors may be a therapeutic option for reducing cytotoxic oedema after ischaemic stroke.

The potential of AQPs as a therapeutic target for reducing oedema has been demonstrated in several studies [109,115,116]. For example, work by Kourghi et al. [117] using in vitro models of choroid plexus and oocytes demonstrated the potential of several loop-diuretic derivatives to inhibit AQP1 and 4 functions. In addition, treating mice subjected to stroke with the loop diuretic bumetanide led to reduced infarct volume and ipsilateral hemispheric water content, as well as a significant reduction in AQP4 protein expression compared with untreated animals [118,119]. In addition to the loop diuretics and their derivatives, antiepileptic drugs and other small molecule inhibitors such as tetraethylammonium [120] and TGN-020 [121] have been shown to inhibit water transport, but with limited progress. Nevertheless, no drug has been approved to target AQPs to reduce brain oedema in stroke. Recently, Sylvain et al. [122] have shown that an FDA-approved drug, trifluoperazine, can reduce cerebral oedema during the early acute phase in mice subjected to photothrombotic stroke. Trifluoperazine is a phenothiazine derivative and a dopamine antagonist with antipsychotic and antiemetic activities, but also inhibits AQP4 expression at both gene and protein levels, and leads to an increase in glycogen levels, suggesting trifluoperazine treatment can be beneficial for brain energy metabolism [122]. Treatment with trifluoperazine inhibited AQP4 localisation to the blood–spinal cord barrier, and led to a reduction in CNS oedema and the acceleration of functional recovery in a rat spinal cord injury model [66].

Several previous studies have also demonstrated that changes in astrocyte AQP4 localisation/polarisation can result in responses to ischaemia and other insults [123,124,125,126], which may not be accompanied by any change in AQP4 expression levels [112,123,127]. This change in AQP4 polarisation might be a potential protective mechanism that counteracts early oedema formation to minimise brain damage [125]. Therefore, inhibiting changes in AQP4 localisation might be a better therapeutic strategy than complete blocking of AQP4, which has an important role in brain fluid homeostasis.

## 5. The Role of Astrocyte Activation in Neuroinflammation after Ischaemic Stroke

Microglia and astrocytes are the major immunocompetent cells of the CNS, although astrocytes are frequently overlooked in this regard [53]. Astrocytes contain a number of receptors that are involved in innate immunity, such as Toll-like receptors (TLR), nucleotide-binding oligomerisation domains, double-stranded RNA-dependent protein kinase, scavenger receptors, mannose receptor and components of the complement system [128]. Both complement component C1q and C3 are associated with astrocyte A1 phenotype [129]. Complement peptide C3a promotes astrocyte survival in response to ischaemic stress, and the protective effect can be reversed by C3a-receptor deficiency [130]. Astrocytes can modulate immune response by inhibiting T cells and monocyte activation [131]. After ischaemic stroke, some reactive astrocytes transform into nonprofessional phagocytes and “clean up” the infarct area [132,133].

Both microglia and astrocytes respond to damage-associated molecule patterns (DAMPs) in ischaemic stroke [134]. DAMPs elicit a strong inflammatory response by activating pattern recognition receptors, such as TLR2 and TLR4 that are crucial inflammatory mediators after stroke. Through activating TLR4, MMP9 is upregulated in neurons and astrocytes [135]. TLR2 plays a detrimental role in the haemorrhagic mouse brain by activating MMP9 in astrocytes, compromising the BBB and enhancing neutrophil infiltration and proinflammatory cytokine gene expression [136]. Suppression of TLR2 and TLR4 reduces nuclear factor-kappa B (NF-κB) activity, which is involved in proinflammatory and redox-active pathways [137]. NF-κB signalling pathway regulates the secretion of cytokines and chemokines in astrocytes under physiological and pathophysiological conditions. The commonly produced cytokines by active astrocytes are interleukins (IL-6, IL-10 and IL-1β), IFN-γ and transforming growth factor β (TGF-β). Studies have previously pointed out that depending on the extent of ischaemic injury, the cytokines secreted by astrocytes may be neurodegenerative or neuroprotective [56]. TGF-β was seen to protect neurons against excitotoxicity by inhibiting the tissue plasminogen activator (tPA) potentiated NMDA-induced neuronal death through a mechanism involving the upregulation of the type-1 plasminogen activator inhibitor (PAI-1) in astrocytes. Additionally, TGF-β was reported to prevent neuronal apoptosis via ERK1/2 pathway [56,57]. The inhibition of the NF-κB pathways leads to reductions in the expression of proinflammatory genes, such as TNF-α, vascular cell adhesion molecule 1 (VCAM-1) and intercellular adhesion molecule 1 (ICAM-1), and the infiltration of CD11b+ leukocytes [138]. Glycolysis induced by tumour suppressor protein 53 reduces the degradation of IκBα and inhibits NF-κB translocation in astrocytes, thereby ameliorating neuroinflammation [139]. Ischaemic preconditioning stimuli ameliorate the inflammatory response after stroke via the TLR/cytokine pathway [140]. Reactive oxygen species (ROS) formation after ischaemic stroke activates signal transducer and activator of transcription 3 (STAT3). A ROS scavenger dimethylthiourea has been shown to inhibit the activation of the STAT3 pathway and attenuates neuroinflammatory and neuronal injury [141]. In addition, the activation of Notch-1 pathway after stroke facilitates the proliferation of reactive astrocytes and restricts infiltration by immune cells [142].

The local inflammation leads to the upregulation of adhesion molecules such as VCAM-1, ICAM-1 and P-selectin in endothelial cells, and the secretion of MMPs such as MMP2 and MMP9 [143,144]. The enhanced expression of adhesion molecules in brain endothelial cells attracts leukocytes and platelets, and promotes the tethering/rolling, firm adhesion and transmigration of these cells across the BBB [145]. Adhesion of leukocytes causes further damage due to the activation of signalling pathways and the release of ROS and inflammatory cytokines, and MMPs from other cells of the NVU. This creates a proinflammatory environment, which further activates the endothelium and leads to increased BBB permeability (Figure 2). This increased permeability due to inflammation has been demonstrated using magnetic resonance imaging (MRI) at both the acute and chronic phases of stroke in patients with ischaemic stroke [146,147] and in animal models [148,149].

Transcriptome analyses show that reactive astrocytes after ischaemic stroke exert both proinflammatory and neuroprotective functions. Zamanian et al. [150] found that *Lcn2* (which may directly promote neuronal death) was induced 228-fold, and *Serpina3n* was induced 9.1-fold in reactive astrocytes one day after experimental ischaemic stroke. Rakers et al. [151] also found that markers of reactive astrocytes, *Lcn2, GFAP, vimentin* and *Timp1,* were highly expressed, and contribute to inflammation (e.g., *Spp1, Cd52, Lcn2* and *Ifi202b),* cell division and migration (e.g., *Cdk1, Myo1f* and *Anxa3*). A2-specific transcripts were intriguingly predominant at 72 h after transient MCAO [151]. Furthermore, reactive astrocytes produce and release proinflammatory mediators (e.g., IL-6, TNF-α, IL-1α, IL-1β and IFN-γ), and free radicals (e.g., NO, superoxide, peroxynitrite), which can lead to neuronal death, infarct progression and increased BBB permeability [19].

## 6. Astrocyte Activation and Glial Scar Formation

The activation of astrocytes in stroke highlights the climax of structural and chemical modifications that act together to form inveterate scars. Elevated synthesis of GFAP and other complementary filaments such as nestin and vimentin occurs at the site of injury in cerebral ischaemia [152]. TGF-β signalling is increased in astrocytes, which regulates glial scar formation and the immune response to stroke [153]. A glial scar is formed around dying brain tissue after ischaemic stroke, which consists predominately of reactive astrocytes, microglia and ECM. Prominent biochemical components of the gliotic scar include secreted macromolecules such as chondroitin sulfate proteoglycans (CSPGs), e.g., aggrecan, brevican, neurocan and phosphacan [154]. Astrocytes in acute lesions upregulate CSPGs, whereas astrocytes in chronic multiple sclerosis lesions express high levels of a protease called “a disintegrin and metalloproteinase with thrombospondin motifs” (ADAMTS) that metabolises CSPGs [155].

The scar has traditionally been viewed to inhibit neurite outgrowth and axonal regeneration. The characteristic of growth inhibitory factor secretion by reactive astrocytes serves as a hindrance to axonal extensions, which deters CNS function recovery at a chronic stage [156,157]. A number of studies consider glial scar formation and reactive gliosis as maladaptive responses; therefore blocking its formation may be beneficial. Targeting glial scar formation or reactive gliosis occurs as an interesting strategy for the treatment of stroke and other neurological disorders. Administration of lipoic acid averts the formation of the glial scar, and promotes angiogenic effects as a vital mechanism for neural rejuvenation [158]. Cyclosporine A (CsA), the most widely used immunosuppressive agent, has been shown to be neuroprotective due to significantly reducing astrogliosis and glial scar formation rather than reducing infarct volume in a rat model of stroke [159].

Nevertheless, reactive gliosis and glial scar formation can serve as neuroprotection in certain stages and regulate neural plasticity inclusive of axonal sprouting, neuron generation, synapse control and function; and maintain CNS homeostasis and restrict neuroinflammation [160]. In addition, glial scar formation prevents the secondary degeneration that occurs at the underlying site of injury where the damage extends to the surrounding environment [161].

## 7. The Role of Astrocyte Secretions in Ischaemic Stroke

Astrocytes are highly secretory cells in the CNS [162]. By secreting neurotrophic factors such as secreted protein acidic and rich in cysteine (SPARC) family proteins and the Hevin glycoprotein, astrocytes promote growth and survival of neurons, control synapse formation and regulate synaptogenesis [25,163]. Other neurotrophic factors such as NGF, BDNF, NT-3, EPO, VEGF, EGF, IGF, GDNF and bFGF secreted by astrocytes promote neuorestoration following stroke (Section 2) [56,57]. Netrins, ephrins and semaphorins are astrocyte-derived proteins that are known to govern axon guidance [25]. Astrocytes also secrete matrix proteins such as connective tissue growth factor (CTGF)/CCN family proteins, MMP family proteins and tenascins. CCN proteins regulate a broad range of cellular activities, such as adhesion, migration and proliferation [19,163]. MMPs are involved in the degradation of ECM, and are engaged with neuronal injury [25,163]. Many components of iron transport are observed in the astrocyte secretome, including transferrin, hephaestin and ceruloplasmin, thereby regulating iron homeostasis [164,165].

The brain has the second-highest expression level of apolipoprotein E (ApoE), which is a well-reported astrocyte secreted protein and is primarily synthesised locally by astrocytes [166,167]. ApoE was originally known to be involved in lipid transport, but also is reported to modulate neurotransmitter release/sequestration, and regulates brain homeostasis [168]. APOE4, but not APOE3, activates the CypA-MMP9 pathway, and may lead to accelerated BBB breakdown causing neuronal and synaptic dysfunction [169,170]. In APOE4 carriers, BBB breakdown contributes to cognitive decline independent of Alzheimer’s disease pathology [170].

The close proximity and the ability of astrocytes to secrete soluble factors allow them to induce BBB phenotype in brain endothelial cells [171]. Astrocytes have been shown to release VEGF, GDNF, bFGF and angiopoietin-1, which influence BBB function. For example, most of these factors are usually added to endothelial growth media in culture to induce BBB phenotype in brain endothelial cells [172]. Therefore, most in vitro BBB models use astrocytes in co-cultures to take advantage of the astrocyte-secreted factors to increase the tightness of brain endothelial cells-characterised by measurement of the transendothelial electrical resistance (TEER), or by assaying paracellular permeability markers across the monolayer [173,174]. Astrocytes are a source of MMPs and VEGF that damage the BBB after ischaemia [175,176]. Other chemical mediators released by astrocytes, such as sonic hedgehog protein (Shh), GDNF, prostaglandins, NO and AA, also regulate tight junctions, blood vessel diameter and blood flow [177,178]. Astrocytes also secrete cytokines and chemokines, such as IL-6, IL-10, IL-1β, IFN-γ and TGF-β. These cytokines can be either neurodegenerative or neuroprotective (Section 5).

Dhandapani et al. [179] reported that soluble factors in astrocyte-conditioned medium (ACM) protect murine neurons from serum-deprivation induced cell death by releasing TGF-β, which activates the activator protein-1 (AP-1) protective pathway and prevents apoptosis. A study has also reported that ACM constituents such as interleukins (IL-6, IL-10 and IL-1β) and TGF-β play vital roles in ACM-induced ischaemic tolerance in neurons [180]. Song et al. [181] demonstrated that ACM exerts neuroprotective effects in ischaemic stroke, through modulation of NT-3, GDNF and TNF-α secretion. Another study reported that ACM provides a neuroprotective effect by regulating apoptosis-related protein expression [182]. Overall, the results from these studies suggest that important factors secreted by astrocytes are vital for neuronal protection during ischaemic injury.

On the other hand, the secretory activity of reactive astrocytes can exacerbate tissue injury. For example, an increased concentration of TNF-α can inhibit neurite outgrowth [183]. Astrocytes also release several pro-inflammatory cytokines (TNF-α, IL-1β, IL-6 and IFN-γ) in response to acute ischaemia, which trigger the production of secondary mediators such as AA metabolites, NO, ROS and MMPs that promote neuronal degeneration and axonal demyelination [56]. The factors secreted by astrocytes can act in an autocrine/paracrine fashion, thereby resulting in amplification of secretion, contributing to sustained astrogliosis and neurotoxicity [184]. Activated astrocytes also secrete compounds with potentially direct toxic effects on neurons/axons and oligodendrocytes/myelin, such as reactive oxygen and nitrogen species, glutamate and ATP [185].

In conjunction with soluble proteins, ACM contains extracellular vesicles (EVs), which can be broadly divided into exosomes (less than 200 nm), microvesicles (200–1000 nm) and apoptotic bodies (larger than 1000 nm). Exosomes are being widely pursued due to their abilities to cross the BBB and interact with target cells [186]. Studies suggest that cells take up exosomes by endocytosis via receptor-mediated adhesion, direct fusion, or via ligand-receptor interactions. Exosomes may contain proteins, lipids, metabolites, miRNA/sRNA, DNA, enzymes, growth factors and cytokines depending on their origins and targets [187]. A study reported that preconditioning neurons with exosomes obtained from glial cells were protective during acute ischaemia [188]. It has been reported that glial cells transfer miRNA to neurons via exosomes targeting signalling pathways such as PI3K/AKT pathway, Hippo, MAPK or mTOR. Exosomal content (such as miRNA) is reported to promote neurogenesis, axonal remodelling, vascular remodelling, and to reduce neuroinflammation [187]. Pei et al. [189] reported that astrocyte-derived exosomes suppressed autophagy and ameliorated neuronal damage during ischaemic stroke. Another study by Hira et al. [190] reported that astrocyte-derived exosomes treated with semaphorin 3a inhibitor enhanced stroke recovery via prostaglandin d2 synthase. Taylor et al. [191] reported that in response to oxidative stress, cultured astrocytes released elevated amounts of heat-shock protein 70 (HSP70) and synapsin 1 in association with exosomes. miR-92b-3p released from astrocytes subjected to oxygen glucose deprivation (OGD) was associated with activation of the PI3K/AKT pathway and ameliorated OGD-induced injury in neurons [192]. Viral vectors expressing miR-124 were found to increase neurogenesis and to promote neuroprotection against cerebral ischaemia in vivo [193]. Astrocytes also release the miR-17-92 cluster that promotes neurite elongation [194]. Taken together, emerging evidence suggest that extracellular vesicles are an important part of the astrocyte secretome and can have important therapeutic implications for ischaemic stroke [195].

## 8. Targeting Astrocytes as a New Therapy for Stroke

The aim of astrocyte targeted strategies is to maximise the potential for astrocyte survival following ischaemic stroke, which may lead to an increase in neuronal survival in the penumbra, essential for favourable post-stroke outcomes [196]. Several promising targets have been identified in preclinical studies that could increase astrocyte survival. For example, overexpressing heat HSP72 and dismutase 2 can increase astrocytes’ resistance under ischaemic stress [52], and ceftriaxone, an antibiotic, has been shown to upregulate expression of GLT-1 in astrocytes, leading to neuronal protection in stroke [197,198]. Furthermore, Deng et al. [199] showed bone marrow-derived mesenchymal stem cell (BMSC)-derived exosomal miR-138-5p promoted the proliferation of mouse primary astrocytes after OGD. BMSC-derived miR-138-5p delivered to astrocytes via exosomes alleviated neuron injury in mice following MCAO.

S100β, a calcium dependent protein which is produced primarily by astrocytes in the CNS, is released into the serum and CSF 24–96 h after ischaemic stroke onset [200,201]. S100β can be neuroprotective at low concentrations but can lead to neuron and astrocyte death at high concentrations [201]. It has been proposed that targeting astrocytes to reduce S100β level could be neuroprotective [202]. Arundic acid (ONO-2506), which reduces the synthesis of S100β in astrocytes [203], has been shown to decrease stroke volume and improve functional outcomes [204]. The protective effect of the arundic acid was greater when administered 24 h after the stroke onset compared with immediate administration [204]. Based on these results, Pettigrew et al. [55] conducted a multi-centre, dose-escalating, randomised, double-blind, placebo-controlled phase 1 trial to examine the effect of arundic acid in ischaemic stroke patients, and showed increasing the infusion of arundic acid in multiple-dose can reduce S100β protein level in serum after acute stroke. However, clinical trials have not been able to show efficacy of arundic acid in stroke [202].

As reviewed by Liu and Chopp [19], in addition to affecting cell survival, astrocytes contribute to angiogenesis, neurogenesis, synaptogenesis and axonal remodelling, leading to improved function in the days to weeks following stroke. Astrocytes can be targeted in these periods to improve stroke rehabilitation, thereby making a significant contribution to improving stroke recovery.

Several studies have suggested that genetically modified astrocytes can be used as a therapeutic target based on their regulation of proteins relevant to immune response and cytotoxicity. For example, Tau hyperphosphorylation (a neurodegeneration hallmark and is closely linked to cognitive function deficiency) occurs following focal brain ischaemia [205]. Therefore, proteins that control microtubular mounts (e.g., glycogen kinase 3 (GSK3) and cyclin-dependent kinase 5 (CDK5)) and remodelling of the actin cytoskeleton can be potential targets for neuroregeneration following stroke [206]. In the CDK5 scenario, Gutiérrez-Vargas et al. [207] designed a CDK5-targeted shRNAmiR (a technique known as RNAi, RNA interference) that contributed to improvement in neurological and motor function in a cerebral ischaemia model in the first week following ischaemia. A month after ischaemia, deterioration of learning, memory and reversal learning was prevented by CDK5 RNAi [208]. Moreover, CDK5 RNAi induced astrocyte stellation and the release of BDNF in a Rac1- dependent manner that provided neuroprotection in astrocyte and neuron co-cultures [208]. Following up on this work, Becerra-Calixto and Cardona-Gómez [206] showed that one month after transplantation of CDK5 knock-down astrocytes into ischaemic rats, motor function recovery and endogenous astrocyte branching around blood vessels were increased, accompanied by increased expression of endothelial PECAM1 and cell proliferation marker, Ki67 in the subventricula zone. These effects were sustained for four months and helped prevent neuronal and astrocyte loss, which supported BBB recovery through secretion of BDNF by endogenous astrocytes in ischaemic rats [209]. Taken together these studies suggest that knockdown of CDK5 in astrocytes could be a potential therapeutic target to protect the BBB and enhance the recovery of the NVU and improve brain function following ischaemic stroke.

It has recently been reported that genetic manipulation can convert astrocytes into functional neurons [210]. Differentiation of stem/precursor cells towards neuronal fate is driven by miR-124 inhibition of REST (a transcriptional repressor of neuron-associated genes, including miR-124), an induction loop that is suppressed by polypyrimidine tract-binding protein (PTB). Downregulation of PTB removes this suppression (including in differentiated cells, e.g., fibroblasts), and contributes to specification of a neuronal cell fate. Depletion of PTB in astrocytes, even transient suppression by lentiviral delivery of shRNA, generated neurons [210]. In a model of Parkinson’s disease, midbrain astrocytes were converted into dopaminergic neurons [210]. There may be therapeutic opportunities in exploiting the prevalence of astrocytes in lesions following ischaemic stroke (due to greater survival and proliferative responses), by converting some astrocytes into neurons, although conversion efficiency reportedly varied across brain regions [210].

Neural precursor cell (NPC) transplantation is another potential option for improving stroke recovery. NPC transplantation in mice led to enhanced functional and structural plasticity up to 60 days post ischaemia. This relied on the ability of transplanted NPCs to localise in the penumbra to help promote GLT-1 upregulation in astrocytes and reduction of peri-ischaemic extracellular glutamate [211]. Secretion of VEGF by NPCs was required for the upregulation of GLT-1, as blocking VEGF during the first week after stroke reduced this upregulation and long-term behavioural recovery of these mice [211].

Furthermore, Luo et al. [212] has shown that co-transplantation of astrocytes with neural stem cells (NSCs) leads to increased survival and neuronal differentiation of NSCs following ischaemic stroke in rats compared with NSC transplantation alone. Therefore, astrocyte co-transplantation with NSC could be a novel strategy for repairing the brain following stroke injury.

Given the important role played by astrocytes in ischaemic stroke, models which can accurately mimic the NVU are essential for furthering our understanding of the mechanisms of astrocyte activation and BBB dysfunction to help develop new therapeutics. While traditional BBB models, which usually comprise of co-cultures of brain endothelial cells with astrocytes in static conditions, can give some important mechanistic information on astrocyte-endothelial interactions, advanced 3D or perfusion-based models are much more suited for investigating the complex molecular and cellular interactions of the NVU during ischaemic conditions [174,213]. Furthermore, these BBB models can give real-time information on permeability changes during ischaemia and release of inflammatory mediators as well as live imaging of the NVU/BBB. For example, perfused NVU/BBB-on-a-chip [214,215,216], dynamic in vitro BBB models [217,218] and 3D multi-cultures/organoids [219,220] can simulate the physiological characteristics of the NVU/BBB, and therefore can be used to model hypoxic/ischaemic conditions to understand the underlying pathophysiological mechanisms. Furthermore, these models can also assess/screen the potential of novel drug candidates and BBB penetration before confirmatory studies in animals, thereby reducing the use of animals, time and resources during the early stages of drug discovery.

## 9. Conclusions

Astrocytes are critical for BBB reconstruction and neuroprotection in the acute stages of ischaemic stroke, as they increase the uptake of extracellular glutamate and sodium/potassium-ATPase activity. Neurotrophic factors released by astrocytes facilitate neurological recovery in the chronic stages. Nevertheless, astrocytes form glial scars, which hinder axon regeneration. In addition, reactive astrocytes produce and release proinflammatory mediators (e.g., IL-6, TNF-α, IL-1α, IL-1β and IFN-γ), free radicals and neurotoxic molecules. Since a large number of astrocytes survive after stroke, and neurons cannot survive if neighbouring astrocytes are lost, astrocytes must be considered as an important treatment target for stroke. Understanding the signalling pathways and molecules secreted by astrocytes in cerebral ischaemia can help guide the development of novel neuroprotective treatments for ischaemic stroke. Indeed, preclinical studies have shown the potential of several target molecules to induce the survival of astrocytes, and reduce cerebral oedema, thereby improving stroke outcomes. Furthermore, genetically modified astrocytes, NPC and NSC have shown promise in experimental strokes by promoting neurorestoration. Furthermore, advanced in vitro models such as perfusion-based NVU/BBB models or organoids can give valuable information on the roles of astrocytes during hypoxic/ischaemic conditions, and screen potential therapeutic candidates for ischaemic stroke.
